# Cossham Medical Society

**Published:** 1982

**Authors:** Peter Moffit


### Cossham Medical Society

**Peter Moffit**

The oldest Bristol Medical Club began its 1981/82 session with an informal presidential ramble. The only message that seemed to emerge was the ease with which the speaker seemed able to lose his way; in the Alps, the Lakes, or even more alarmingly, a selection of dirty wet caves in Somerset and Wales. The photographs, however, were sufficiently stimulating to produce further tall stories at the bar afterwards. As an interlude, the president's daughter provided evidence of more competent navigation on a solo wander through the Norwegian hills.

November provided the sad swan-song of the Radiologist Dr. Mackenzie-Crooks. Punctuated by travel slides, some of them predictably good-looking, the speaker gave a historic and mildly cynical account of the advance of imaging techniques, which did not altogether claim that his department could provide all the answers. Despite nuclear-this, and double-contrast-that, it seems that thorough clinical examination still has a place. David has since wandered off goodness-knows-where.

December brought us Dr. Martin Mott, whose talk on Cancer in Childhood described the painstaking work of recent years, combining cytotoxic with synergistic treatments, the constant objective being to find the best. The devotion and attention to detail that have brought many remissions and a few cures were inspiring and humbling.

January 5th seemed a good moment for Dr. Philip Golding to address a penitent audience on what can be done for alcoholics. His experience was more hopeful than some, and details of the centre that he manages were avidly digested. It's on these occasions that we furtively look round at one another and try and guess who's the worst.

It's not really possible to describe what went on at the February meeting, as Dr. Bob Eastham thinks and hypothesizes faster than most of us can listen. Considerately, he punctuated his anthropological and haematological comments with a world-wide series of slides, which left us as breathless at his knowledge. Reference was duly made to his modest and total accessibility for information and advice.

In March, Dr. Martyn Gay aroused us before his arrival by the choice of his title: The Problem with Teenagers are their Parents. With typical humour, and sometimes sadness, he developed his thesis, but surely we can't be held responsible for those awful children we've got – or can we?

The planned season ended in April with a lecture by Dr. Martin Gledhill, a former student of one of us. Attachments have their uses, apart from stimulating G.P.'s to do some rapid secret revision! Martin described some expeditions he'd led in the Mediterranean and off Scotland, finding various goodies from ancient shipwrecks. One of the most fascinating finds was a complete load of roof tiles from early in the first millennium.

An extra summer event, in conjunction with Frenchay Postgraduate Club, was a recital-cum-lecture by Mr. John Marsh in a most luxurious outhouse of Mr. Britton's home at Shortwood. Brilliant organ-playing was combined with humour and refreshments, and not a word of medicine was mentioned.

